# Human ACE2 Polymorphisms from Different Human Populations Modulate SARS-CoV-2 Infection

**DOI:** 10.3390/v14071451

**Published:** 2022-06-30

**Authors:** Pan Hu, Vanessa L. Bauer, Sara L. Sawyer, Felipe Diaz-Griffero

**Affiliations:** 1 Department of Microbiology and Immunology, Albert Einstein College of Medicine, Bronx, NY 10461, USA; pan.hu@einsteinmed.org; 2 BioFrontiers Institute, Department of Molecular, Cellular, and Developmental Biology, University of Colorado, Boulder, CO 80303, USA; vanessa.bauerdumont@colorado.edu (V.L.B.); ssawyer@colorado.edu (S.L.S.)

**Keywords:** SARS-CoV-2, human ACE2, polymorphisms, COVID-19, infection

## Abstract

The COVID-19 pandemic caused by severe acute respiratory syndrome coronavirus 2 (SARS-CoV-2) has resulted in over 6 million deaths worldwide. The high variability in COVID-19 symptoms remains one of the most interesting mysteries of the pandemic. Genetic and environmental factors are likely to be key determinants of COVID-19 symptomatology. Here, we explored ACE2 as a genetic determinant for SARS-CoV-2 infection and COVID-19 symptomatology. Each human genome encodes two alleles of ACE2, which encodes the cell entry receptor for SARS-CoV-2. Here, we determined whether naturally occurring human ACE2 (hACE2) polymorphisms in the human population affect SARS-CoV-2 infection and the severity of COVID-19 symptoms. ACE2 variants S19P, I21V, E23K, K26R, K31R, N33I, H34R, E35K, and T92I showed increased virus infection compared to wild-type ACE2; thus, these variants could increase the risk for COVID-19. In contrast, variants D38V, Y83H, I468V, and N638S showed reduced infection, indicating a potential protective effect. hACE2 variants K26R and T92I increased infection by three-fold without changing the levels of ACE2 on the surface of the cells, suggesting that these variants may increase the risk of severe COVID-19. On the contrary, hACE2 variants D38V and Y83H decreased SARS-CoV-2 infection by four- and ten-fold, respectively, without changing surface expression, suggesting that these variants may protect against severe COVID-19. Remarkably, all protective hACE2 Polymorphisms were found almost exclusively in Asian populations, which may provide a partial explanation for the low COVID-19 mortality rates in Asian countries. Thus, hACE2 polymorphisms may modulate susceptibility to SARS-CoV-2 in the host and partially account for the differences in severity of COVID-19 among different ethnic groups.

## 1. Introduction

The severe acute respiratory syndrome coronavirus 2 (SARS-CoV-2), the etiologic agent of the coronavirus disease 2019 (COVID-19) pandemic [1,2,3], has affected over 500 million people with over 6 million deaths worldwide since the end of 2019 (WHO, June 2022). The symptomatology of this novel illness includes fever, fatigue, myalgia, dry cough, and shortness of breath. In some cases, nasal congestion, sore throat, hemoptysis, lymphocytopenia, and diarrhea are observed [3,4]. High mortality is observed in older patients (>65 years old), especially those with hypertension, diabetes, or renal failure (Centers for Disease Control and Prevention, 2022). SARS-CoV-2 infection may result in fatal pneumonia [3,4,5]. In some individuals, however, SARS-CoV-2 infection is asymptomatic [6]. These discrepancies in the severity of symptoms are difficult to explain; however, individual differences in genetic makeup likely contribute to the variability in COVID-19 symptoms [7]. hACE2 gene polymorphisms vary among ethnic groups; thus, ethnicity is one factor that may predict COVID-19 severity [8].

SARS-CoV-2 binds and infects cells via the human angiotensin-converting enzyme 2 (hACE2) receptor [9,10,11,12,13], using the receptor-binding domain (RBD) of the SARS-CoV-2 spike (S) protein [9,14]. The ACE2 gene is located on the X chromosome, and the protein is expressed on the surface of lung epithelial cells and other tissues. The hACE2 protein regulates the renin-angiotensin-aldosterone system, which controls blood pressure [15]. The 805 amino acid hACE2 protein is a type I cell surface glycoprotein with peptidase activity, anchored to the cellular membrane by its C-terminal domain [16].

Here, we determined the contribution of polymorphisms in the hACE2 gene to the success of infection by SARS-CoV-2. We focused primarily on polymorphisms that affect amino acids at the interface between the spike protein of SARS-CoV-2 and the hACE2 receptor. We determined whether hACE2 variants S19P, I21V, E23K, K26R, K31R, T27A, N33I, H34R, D38V, Y83H, T92I, V184A, S257N, G326E, G352V, I468V, N368S, L656stop, and N720D modulate SARS-CoV-2 infection. Interestingly, some hACE2 variants increased SARS-CoV-2 infection by 2–3-fold, and other variants decreased SARS-CoV-2 infection by 4–10-fold. These results provide evidence that hACE2 polymorphisms modulate SARS-CoV-2 infection, possibly explaining the differences in susceptibility to and severity of COVID-19 among different ethnic groups.

## 2. Materials and Methods

### 2.1. Human ACE2(hACE2) Mutagenesis

The plasmid containing hACE2 was a generous gift from Dr. Massimo Pizzato. Empty vector pScalps-Puro was purchased from Addgene. Plasmids expressing the hACE2 mutants were created by site-directed mutagenesis. Primers were designed using PrimerX (https://www.bioinformatics.org/primerx/, accessed on September 2021). The mutations in all the clones were confirmed by Sanger sequencing (Genewiz).

Primers list:



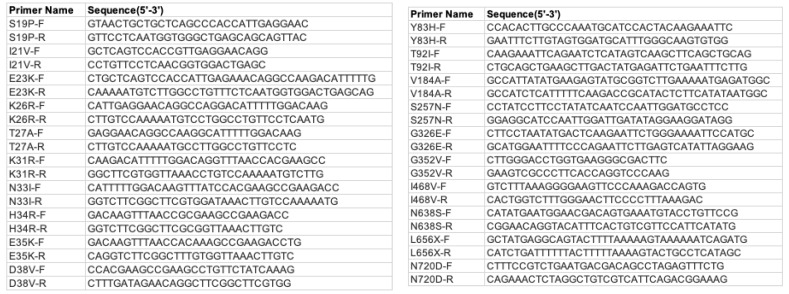



### 2.2. Cell Culture and Virus Production

Human embryonic kidney 293 cells (HEK293) and Vero E6 cells were grown in Dulbecco’s modified Eagle’s medium (DMEM) supplemented with 10% fetal bovine serum and 1% penicillin/streptomycin at 37 °C, 5% CO_2_. HEK293(CRL-3216) and Vero E6(CRL-1586) cells were purchased from ATCC(Washington DC). CoV-2_rVSV-GFP_, a chimeric vesicular stomatitis virus expressing the SARS-CoV-2 wild-type spike protein from the Wuhan-Hu-1 strain (GenBank: MN908947) expressing GFP as a reporter of infection, was a kind gift from Paul Bieniasz [17].

Lentiviruses to generate stable cell lines expressing the hACE2 variants were generated by transient transfection of HEK293 cells with 1 μg of HDM (HIV-1 gag-pol of HIV-1 NL4–3), 5 μg of the pScalps-Puro vector containing each hACE2 variant, 1 μg of pVSV-G, 1 μg of Tat (trans-activator of transcription) and 1 μg of Rev (regulator of expression of virion proteins). The medium was changed 12 h post-transfection. Supernatants were harvested at 48 h after transfection.

### 2.3. Production of hACE2 Stable Cell Lines

HEK293 cells were transduced with recombinant lentiviruses containing each hACE2 variant and pScalps-Puro as a negative control. Transduced stable HEK293 cells were selected in 4 μg/mL puromycin (Sigma, St. Louis, MO, USA).

### 2.4. Infections

CoV-2_rVSV-GFP_ chimeric viruses were expanded by infecting Vero E6 cells for 48 h, after which the supernatant was collected and centrifuged for 10 min at 3000× *g*. The virus was titrated and aliquoted. Infections were performed using 5 × 10^4^ of the indicated cells seeded in 24-well plates for 20 hours at 37 °C with CoV-2_rVSV-GFP_ of the indicated multiplicity of infection (MOI). The percentage of GFP-positive cells was measured by flow cytometry using a BD FACSCelesta.

### 2.5. Geographic Allele Frequency Data for hACE2 Variants

Each gene variant functionally tested in this study was found in the dbSNP database (https://www.ncbi.nlm.nih.gov/snp/rs758142853#variant_details, accessed on September 2021) to determine their geographic allele frequencies. We report allele frequency data from the following studies: 1000 genomes, ExAC, gnomeAD-exomes and gnomeAD-genomes.

## 3. Results

### 3.1. Distribution of Human ACE2(hACE2) Polymorphisms in the Human Population

We compiled a list of hACE2 gene polymorphisms from previous reports in the literature. We focused on, but not exclusively, those located in the region of the hACE2 protein that interacts with the RBD of the SARS-CoV-2 spike protein [8,18,19,20,21,22,23] (Table 1).

These polymorphisms resulted in the following protein changes, S19P, I21V, E23K, K26R, K31R, T27A, N33I, H34R, D38V, Y83H, T92I, V184A, S257N, G326E, G352V, I468V, N368S, L656stop, and N720D. To standardize the geographic allele frequency data for each of these polymorphisms, we searched for them in the dbSNP database, and report the allele frequency of each variant on different continents. Some of these polymorphisms are geographically and locally isolated (Table 1). For example, S19P is observed only in Africa, variants I21V, E23K, and T92I only within Europe, and K31R, N33I, H34R, D38V, Y83H, V184A, S257N, N638S, and L656stop are observed only in Asia. On the other hand, I468V is observed in African, European, and Asian populations, and K26R and N720D are found on all continents.

### 3.2. Location of Human Polymorphisms in the hACE2 Structure

**Figure 1:**
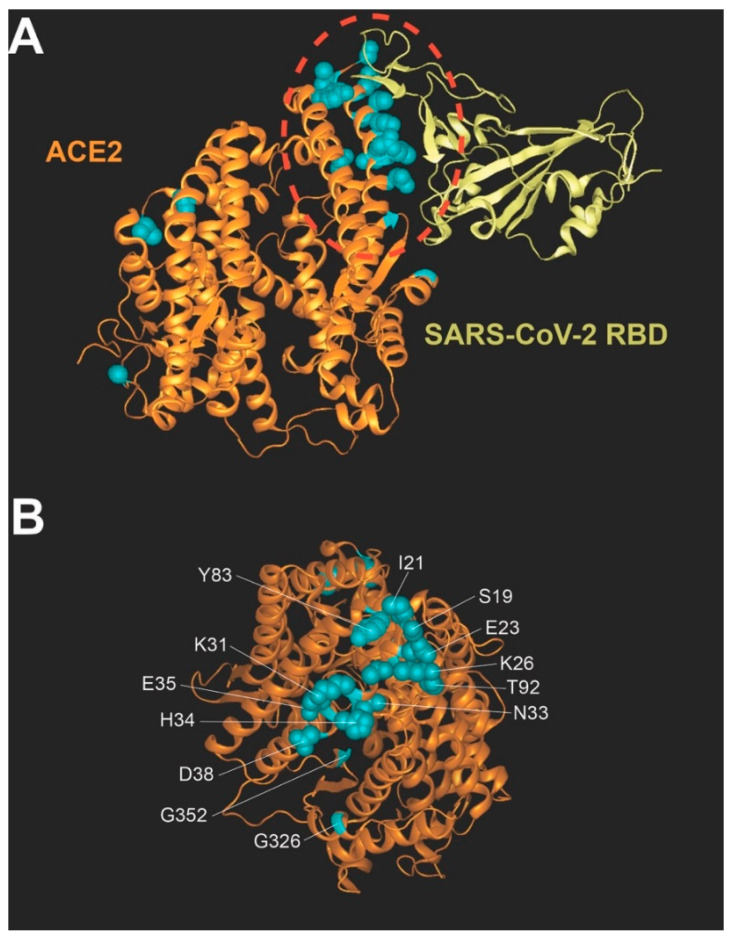
Localization of polymorphisms in the human ACE2 structure. (**A**) The structure of the hACE2 receptor (orange) in complex with the receptor-binding domain (RBD) of the SARS-CoV-2 spike (yellow) (6m0j). The interface between human ACE2 and the spike protein is circled in red, and the location of hACE2 polymorphisms are shown in cyan. (**B**) hACE2 residues at the interface between ACE2 and the spike are shown, forming a cluster in the center of the structure. hACE2 polymorphisms are shown in cyan.

Amino acid residues of hACE2 that are affected by the polymorphisms shown in Table 1 are shown in cyan (Figure 1A,B) in the overall structure of hACE2 (orange in Figure 1A) complexed with the RBD of the SARS-CoV-2 spike protein (yellow in Figure 1A). This research focused on polymorphisms that are localized to the hACE2 region that interacts with the RBD region of the spike protein (Figure 1B).

### 3.3. Effect of hACE2 Polymorphisms on Infection by SARS-CoV-2

**Figure 2:**
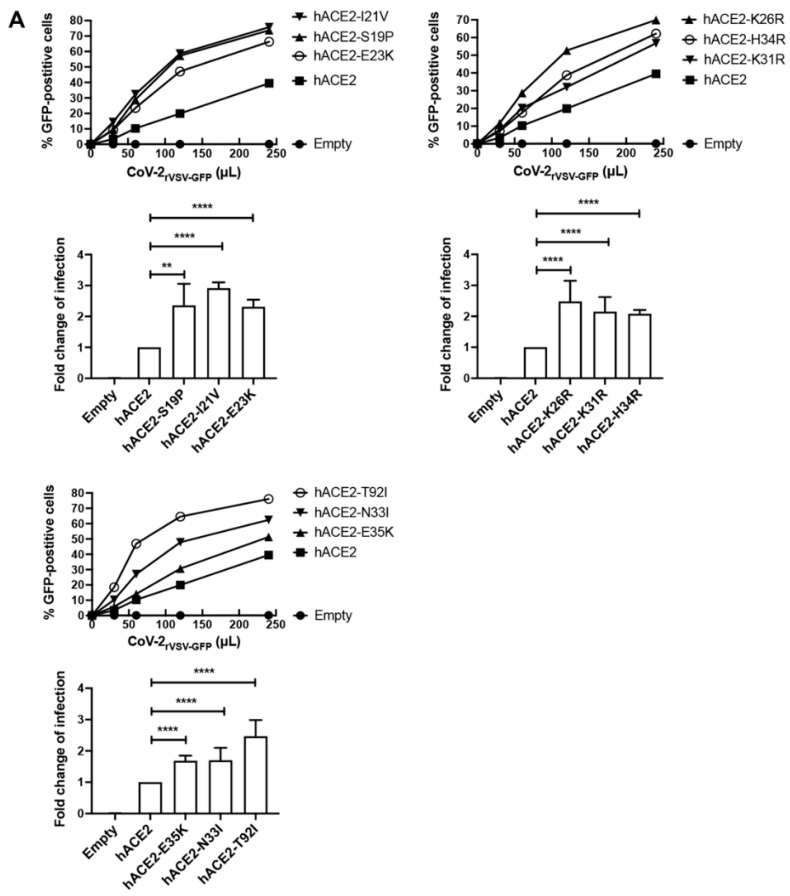

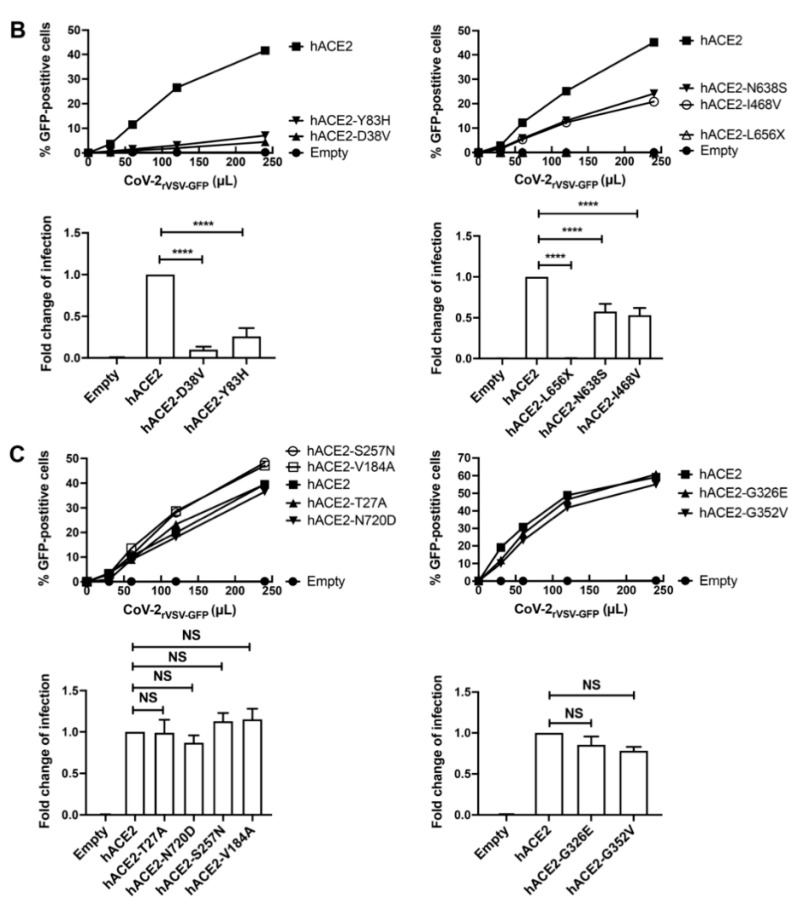

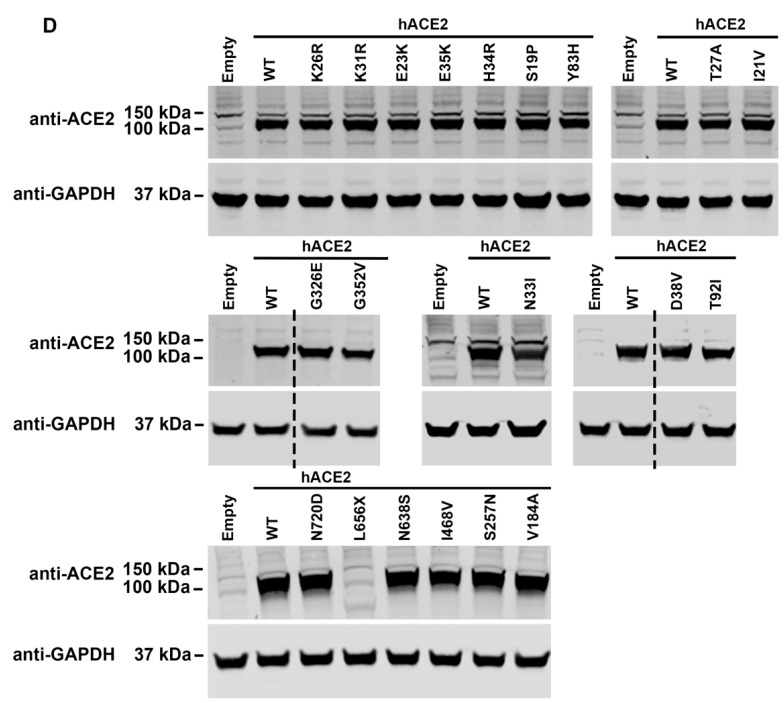
The effect of hACE2 polymorphisms on SARS-CoV-2 infection of cells. (**A**–**C**) Human HEK293 cells stably expressing the different hACE2 variants were infected with increasing volumes of a chimeric vesicular stomatitis virus expressing the SARS-CoV-2_wuhan_ spike-containing GFP as a reporter of infection (CoV-2_rVSV-GFP_). Empty vector pScalps-Puro served as a negative control (Empty). The infection was determined by measuring the percentage of GFP-positive cells 20 h post-challenge. Experiments were performed at least five times, and a representative infection is shown. Statistical analysis was performed using an intermediate value taken from the infection curves (bottom panels). Fold changes for infection with each variant with standard deviations are shown for at least five experiments. ** indicates *p*-value < 0.001, **** indicates *p*-value < 0.0001, NS indicates not significant as determined by using the unpaired *t*-test. (**D**) Expression of hACE2 in HEK293 cells stably expressing the different hACE2 variants was measured by Western blot analysis using anti-hACE2 antibodies. GAPDH was used as a loading control.

Amino acid changes in the hACE2 receptor may increase or decrease the infectivity of SARS-CoV-2; thus, we measured the effect of the different hACE2 polymorphisms on SARS-CoV-2 infection. We stably expressed the hACE2 variants in human HEK293 cells and challenged them with a chimeric vesicular stomatitis virus (VSV) expressing the spike protein of the SARS-CoV-2_wuhan_-containing green fluorescent protein (GFP) (CoV-2_rVSV-GFP_) as a reporter of infection (Figure 2A). As a control, we challenged HEK293 cells containing the empty vector pScalps-Puro.

We identified hACE2 polymorphisms that increased (Figure 2A and Table 2), decreased (Figure 2B and Table 2), or did not change infection by CoV-2rVSV-GFP (Figure 2C and Table 2) compared to wild-type hACE2. hACE2 variants S19P, I21V, E23K, K26R, K31R, H34R, and T92I increased infectivity 2–3-fold while variants N33I and E35K increased infection by less than 2-fold (Figure 2A and Table 2). Interestingly, all changes that enhanced infectivity by 2–3-fold are in proximity in the structure (Figure 1B), suggesting that changes in this region of the receptor have the potential to increase infectivity. These polymorphisms could increase susceptibility to SARS-CoV-2 infection and the severity of COVID-19 in the populations where they segregate. hACE2 variants D38V and Y83H potently decreased infection of SARS-CoV-2 by 4- and 10-fold, respectively (Figure 2B and Table 2), suggesting that these polymorphisms may be protective against SARS-CoV-2 infection and could mitigate COVID-19 severity. hACE2 variants T27A, V184A, S257N, G326E, G352V, and N720D had no effect on infection by SARS-CoV-2 (Figure 2C and Table 2), producing the same infection results as wild-type. To control for levels of expression of hACE2 variants, we used human cells expressing similar levels of the different hACE2 variants (Figure 2D and Table 2).

HEK293 cells stably transduced with the different hACE2 polymorphisms were analyzed for expression by Western blotting using anti-hACE2 antibodies. As a loading control, samples were also blotted using anti-GAPDH antibodies.

### 3.4. Cell Surface Expression of hACE2 Polymorphisms

**Figure 3:**
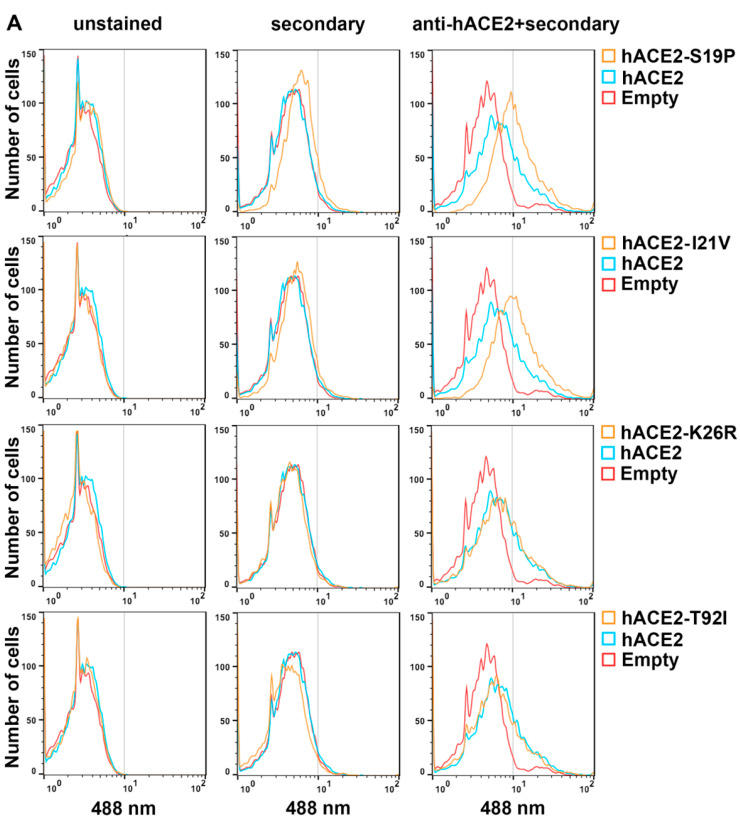

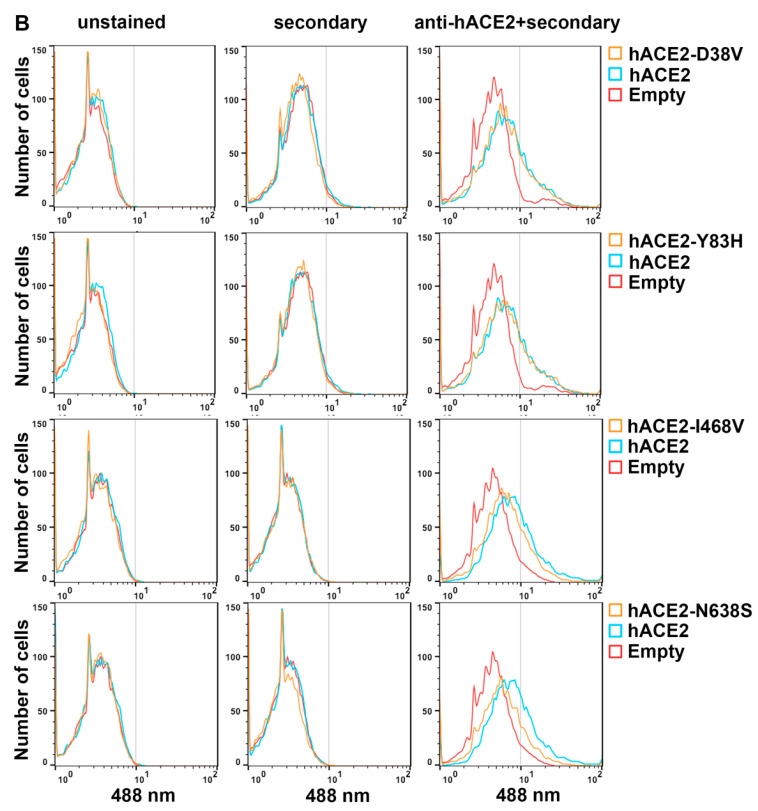
Surface expression in stable cells expressing hACE2 variants. (**A**,**B**) HEK293 cells stably expressing the different hACE2 variants were analyzed for hACE2 surface expression using flow cytometry using anti-hACE antibodies (unpermeabilized cells). Controls were mock stained or received only secondary antibodies. A total of 10,000 events were recorded. The red population indicates cells transduced with the Pscalps-Puro empty vector. Experiments were repeated at least three times, and representative histograms are shown.

Since we measured total hACE2 protein in HEK293 cells expressing the different hACE2 variants, different amounts of cellular protein expression could not account for the differences in infection among hACE2 variants (Figure 2D). Because differences in the amount of hACE2 receptor could cause changes in infectivity, we measured the hACE2 receptor on the surface of the cell by flow cytometry using antibodies against hACE2. The S19P and I21V hACE2 variants that increased SARS-CoV-2 infection were more highly expressed on the surface than wild-type hACE2 (Figure 3A and Table 2), perhaps explaining their increased infectivity (Figure 2A).

Intriguingly, hACE2 variants K26R and T92I showed similar surface expression to wild-type hACE2 (Figure 3A), suggesting that increased binding to the spike protein of SARS-CoV-2 or different properties of these variants led to increased infection. The I468V and N638S hACE2 variants that decreased SARS-CoV-2 infection showed decreased expression on the cell surface compared to wild-type hACE2 (Figure 3B and Table 2), which may explain the decrease in infectivity. Interestingly, hACE2 variants D38V and Y83H showed similar cell surface expression to wild-type hACE2 (Figure 3B and Table 2), suggesting that the decrease in infectivity is due to decreased binding to the spike protein of SARS-CoV-2 or that another property of these variants is defective. Thus, four hACE2 variants had the same level of cell surface expression as the wild-type hACE2 but modulated SARS-CoV-2 infectivity.

## 4. Discussion

According to the data shared by The Johns Hopkins Coronavirus Resource Center (https://coronavirus.jhu.edu/, accessed on January 2022), among the most affected countries, Brazil had the highest COVID-19 mortality rate with 313.78 deaths per 100,000 populations (5 June 2022). Interestingly, European countries, including Italy, Spain, France, Germany, and the UK, had mortality rates in the range of 167–277 deaths per 100,000 populations. By contrast, Asian countries such as China, South Korea, Mongolia, and Japan displayed lower mortality. China had a mortality rate of 1.03 deaths per 100,000 populations, one of the lowest observed. South Korea displayed 47.33 deaths per 100,000 populations, Japan displayed 24.33 deaths per 100,000 populations, and Mongolia displayed 66.47 deaths per 100,000 populations. Overall, these data suggest that Asian countries have lower deaths per 100,000 populations when compared to Europe and the United States.

Here, we determined whether hACE2 polymorphisms found in different human populations can modulate infection by SARS-CoV-2, impacting the susceptibility and severity of COVID-19. Several of the polymorphisms in this study were previously characterized for their ability to bind to the spike protein of SARS-CoV-2 by binding assays [24] or by molecular modeling [8,20,23,24,25]. Several polymorphisms were also characterized using an infectivity assay in cells transiently expressing ACE2 variants that did not reveal any differences [22].

We found that hACE2 polymorphisms K26R and T92I increased SARS-CoV-2 infectivity by 2–3-fold without affecting cell surface expression. Molecular modeling studies predict that hACE2-K26R protein binds to the spike protein with higher affinity than does the wild-type [8,20,25,26], which is supported by the increased affinity of K26R for the spike protein compared to the wild-type based on an ELISA binding assay [24]. Our findings that hACE2-K26R improves infectivity by SARS-CoV-2 are consistent with these prior results and with the finding that increased affinity between hACE2 and the spike protein increases infectivity [27]. An increase in the affinity of viral ligands for their cellular receptors increases infection and disease severity [28,29,30]; thus, the increased affinity supports the infection of cells with low expression of hACE2 by expanding the number of host tissues infected by the virus [30] and could increase infectivity and aggravate COVID-19 symptoms. Interestingly, while hACE2-K26R was found in all populations, it was more common in Europeans.

Like hACE2-K26R, T92I also increased infectivity by SARS-CoV-2 without any changes in the cell surface expression of the protein. Molecular modeling and binding assays showed that hACE2-T92I binds with a higher affinity to the spike protein compared to wild-type hACE2 [24]. Interestingly, T92I targets the glycosylation motif 90NXT92, and previous studies that targeted ACE2 glycosylation by mutation of residues N90 or T92 showed increased affinity between ACE2 and the SARS-CoV-2 spike protein [29,31,32]. Our results showing increased infectivity are consistent with the evidence that hACE2-T92I has a greater affinity for the spike protein than does the wild-type hACE2. hACE2-T92I, which has been found only in the European population, may increase the severity of COVID-19. Overall, our results suggest that polymorphisms K26R and/or T92I are risk genetic factors for COVID-19.

We also discovered hACE2 polymorphisms such as D38V that decreased infection by SARS-CoV-2, demonstrating the protective effect of these variants. Molecular modeling and binding assays showed that hACE2-D38V has a lower affinity for the spike protein compared to wild-type hACE2 [24,33], and we showed here that this variant decreased infection by SARS-CoV-2 by ~10 fold. Interestingly, this protective allelic variant is found only in Asian populations (Table 1), while the hACE2-T92I that increased infection was detected only in the European population (Table 1). This may explain the discrepancy in the prevalence and mortality rates of COVID 19 in Europe and East Asia.

hACE-Y83H, also found only in the Asian population (Table 1), decreased SARS-CoV-2 infection by ~4 fold. Similarly, molecular modeling and binding assays showed that hACE2-Y83H binds the spike protein with decreased affinity, consistent with our findings that hACE2-Y83H reduced infectivity. The residue Y83 makes direct contact with F486 on the spike protein, and disrupting this interaction decreases the affinity between hACE2 and the spike protein [24]. Thus, we identified two hACE2 polymorphisms that were protective against SARS-CoV-2 infection. We also identified hACE2 variants that decreased infection and cell surface expression simultaneously. hACE2-I468V and hACE2-N638S decreased infection modestly compared to hACE2-D38V; thus, they could still represent a protective factor for COVID-19.

We identified two hACE2 polymorphisms, S19P and I21V, that increased SARS-CoV-2 infection by ~2–3 fold, and the increase in infection was explained by an increase in hACE2 cell surface expression. Interestingly, hACE2-S19P is found only in the African populations (Table 1), while I21V is found only in the European populations (Table 1). Similarly, we found that hACE2-E23K, K31R, N33I, and H34R also increased infection by SARS-CoV-2, but to a lesser extent.

The hACE2 gene is in chromosome X, which implies that women have a higher allelic diversity for hACE2 when compared to men due to the simple reason that women carry two alleles. Higher allelic diversity increases the chance of possessing a protective allele, if such an allele exists in a particular population. Therefore, this reasoning will hypothetically imply that in any population women should have a higher probability of carrying a protective hACE2 allele.

Although the severity of COVID-19 likely depends on many genetic and environmental factors, we determined the contribution of hACE2 polymorphisms to SARS-CoV-2 infectivity, which has been shown to be directly proportional to COVID-19 severity [28,29,30]. We demonstrated that hACE2 polymorphisms modulate SARS-CoV-2 infection in the host, thereby affecting the severity of COVID-19.

## Figures and Tables

**Table 1:** ACE2 polymorphisms found in different populations.

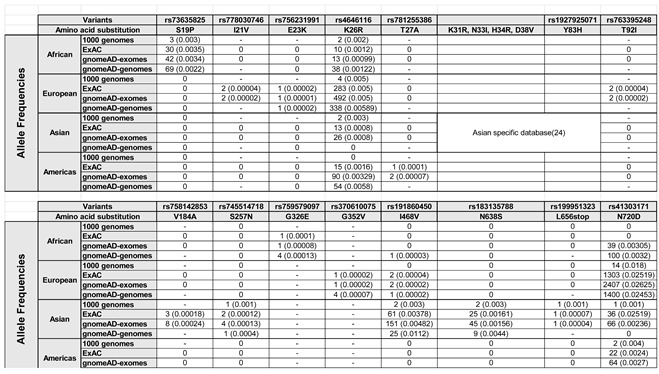

hACE2 genetic variants are named according to the amino acid substitution of the wild type hACE2. Identification numbers registered at the SNP database are listed. Data from the 1000 genomes, ExAC, gnomeAD-exomes, and gnomeAD-genomes were used. -, not found in that genome project. Minor allele counts (frequencies) are shown.

**Table 2:** Phenotypes of hACE2 proteins.

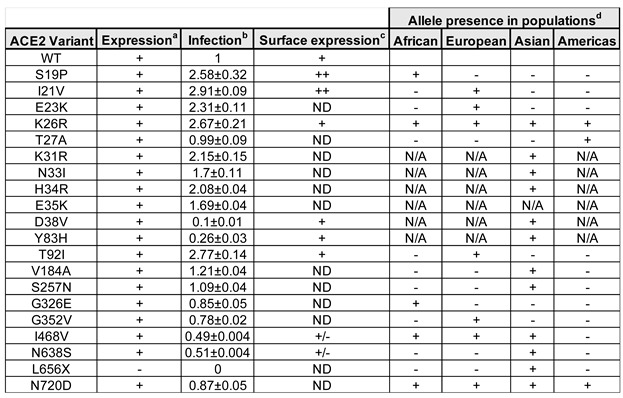

^a^ ACE2 expression was determined by Western blots using anti-hACE2 antibodies, as described in Materials and Methods: +, present; -, absent. ^b^ Infectivity of human cells stably expressing the indicated hACE2 variant was determined as described in Materials and Methods. Fold change of infection for all variants with standard deviations for at least five experiments is shown. ^c^ Surface expression of the hACE2 variants was determined using flow cytometry, as described in Materials and Methods: +, wild-type ACE2 surface expression; ++, higher surface expression when compared to wild-type ACE2; +/-, lower surface expression compared to wild-type ACE2. ND not determined. ^d^ Allele occurrence of specific human ACE2 variants was determined based on data from the 1000 genomes, ExAC, gnomeAD-exomes, and gnomeAD-genomes, as described in materials and methods: +, genetic variant is found in that population; -, genetic variant is not found in that genome project. N/A, not available.

## Data Availability

Publicly available datasets were analyzed in this study. This data can be found here: dbSNP database (https://www.ncbi.nlm.nih.gov/snp/rs758142853#variant_details); The Johns Hopkins Coronavirus Resource Center (https://coronavirus.jhu.edu/); Centers for disease control and prevention (https://www.cdc.gov/nchs/covid19/mortality-overview.htm).
